# Controlling Charge Carrier Dynamics in Porphyrin Nanorings by Optically Active Templates

**DOI:** 10.1021/acs.jpclett.3c03304

**Published:** 2023-12-11

**Authors:** Shrabanti Mondal, Uttam Chowdhury, Subhajit Dey, Md Habib, Carlos Mora Perez, Thomas Frauenheim, Ritabrata Sarkar, Sougata Pal, Oleg V. Prezhdo

**Affiliations:** 1 Department of Chemistry, University of Gour Banga, Malda 732103, India; 2 Department of Chemistry, Sripat Singh College, Jiaganj 742122, India; 3 Department of Chemistry, University of Southern California, Los Angeles, California 90089, United States; 4 Bremen Center for Computational Materials Science, Universität Bremen, Bremen 28359, Germany; 5 Beijing Computational Science Research Center, Beijing 100193, China; 6 Shenzhen JL Computational Science and Applied Research Institute, Shenzhen 518109, China; 7 Department of Physics and Astronomy, University of Southern California, Los Angeles, California 90089, United States

## Abstract

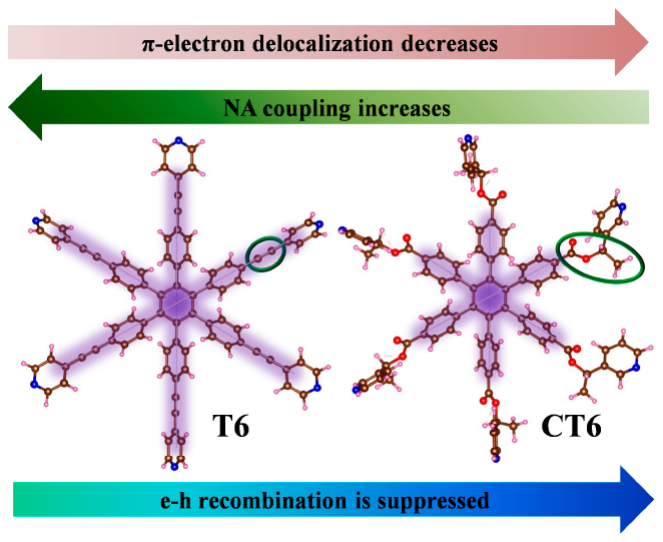

Understanding the dynamics of photogenerated charge carriers is essential for enhancing the performance of solar and optoelectronic devices. Using atomistic quantum dynamics simulations, we demonstrate that a short π-conjugated optically active template can be used to control hot carrier relaxation, charge carrier separation, and carrier recombination in light-harvesting porphyrin nanorings. Relaxation of hot holes is slowed by 60% with an optically active template compared to that with an analogous optically inactive template. Both systems exhibit subpicosecond electron transfer from the photoactive core to the templates. Notably, charge recombination is suppressed 6-fold by the optically active template. The atomistic time-domain simulations rationalize these effects by the extent of electron and hole localization, modification of the density of states, participation of distinct vibrational motions, and changes in quantum coherence. Extension of the hot carrier lifetime and reduction of charge carrier recombination, without hampering charge separation, demonstrate a strategy for enhancing efficiencies of energy materials with optically active templates.

Harvesting and converting sunlight energy into electricity are vitally important for humankind, and a photon absorber is the key component of such a photovoltaic device. Photoexcitation from the valence band (VB) to the conduction band (CB) results in the formation of hot charge carriers (electrons and holes).^1−5^ The carriers lose their energy by cooling to the respective band edges. Extracting photogenerated charge carriers with minimal energy and carrier losses is desirable for efficient device operation. Slow hot carrier relaxation and prolonged carrier lifetimes can enhance the efficiency by reducing charge and energy losses.^6−11^ Hence, the dynamics of carrier cooling is an extremely important factor for practical applications.^12−16^ Spatial separation of the photogenerated charge carriers is another important aspect of a photovoltaic device.^17−20^ Separated carriers can produce photovoltage; alternatively, they can drive photoredox reactions.^21−23^ The dynamics of carrier separation and relaxation can be tuned by various factors, such as material size,^24^ shape,^25^ strain,^26^ orientation,^27^ defects,^28^ doping,^29−32^ temperature,^11^ pressure,^33^ external electric field,^34^ pH,^35^ humidity,^36^ etc.

π-Conjugated porphyrin macromolecular systems are attractive for solar energy applications. They can be used as photosensitizers,^37−39^ charge transporters,^40,41^ and light-harvesting antennae.^42,43^ A fundamental understanding of the excited state properties and charge carrier dynamics in these systems is essential for improving solar cell performance. Previously, we have studied the photoinduced carrier relaxation dynamics of porphyrin systems by considering the influence of varying central metal atoms in the porphyrin units,^44^ the changes in the nanoring geometry,^4^ and the insertion of templates into the nanoring.^45^ Particular attention has been paid to the underlying photophysics to achieve the properties needed for effective device performance.

In this Letter, we demonstrate that optically active templates provide an additional handle for controlling porphyrin properties for photovoltaic applications and that such templates can be used to improve porphyrin performance in artificial light-harvesting systems. Using nonadiabatic (NA) molecular dynamics (MD) in combination with time-domain density functional tight binding theory (DFTB), we investigate the dynamics of photogenerated charge carriers in two porphyrin nanorings composed of hexameric zinc-centered porphyrin unit (Z6) interfaces with optically inactive and active templates involving the acetylenic linker, T6, and the chiral ethyl formate linker, CT6, respectively. We report intraband hot hole relaxation in the subpicosecond to picosecond time regime, which is well corroborated with the experimental findings.^46^ The hot hole relaxation is slower in Z6-CT6 than in Z6-T6 due to the weaker NA coupling between the electronic states in the VB. Photoexcitation of the porphyrin nanorings results in ultrafast electron transfer (ET) from the Z6 core to the templates on a subpicosecond time scale: 520 and 830 fs in Z6-T6 and Z6-CT6, respectively. Charge localization of the donor states and smaller NA coupling between the donor and acceptor states decelerate the ET in Z6-CT6. In addition, we show that the exciton recombines with a 17 ns time constant in Z6-T6. In contrast, Z6-CT6 delays the recombination markedly, by a factor of 6, with the lifetime extended to 102 ns. Hence, longer-lived excitons can be achieved using optically active templates, which weaken the NA electron–vibrational coupling by partial localization of π-electrons, widen the energy gap, separate the electron and hole spatially, and accelerate decoherence at the band edge. The thorough time-domain atomistic investigation of the carrier relaxation dynamics of optically active porphyrin nanorings provides valuable insights into the mechanism of enhancement of photovoltaic performance.

The self-consistent charge density functional tight-binding (SCC-DFTB) method and nonadiabatic molecular dynamics (NAMD) theory^47^ have been applied to investigate the photoexcited carrier relaxation dynamics of the porphyrin macromolecular systems. SCC-DFTB provides reasonable accuracy with minimal simulation cost, allowing one to model large electronic systems. Exploring these large systems with conventional ab initio methods is computationally expensive. Various surface-hopping NAMD methods,^48−53^ along with the classical path approximation (CPA),^54,55^ are available. The present systems of interest are stable at room temperature and do not exhibit any structural deformations, such as reorganization, isomerization, or fragmentation, upon photoexcitation. Hence, the CPA can be successfully applied^56−63^ to treat the heavier nuclear degrees of freedom classically with Newton’s equation of motion. The lighter electrons are described quantum mechanically, and their evolution depends parametrically on the nuclear trajectory. This approach accomplishes NAMD simulation efficiently and cost-effectively. Decoherence has a significant influence on electronic transitions that occur through large energy gaps or between spatially separated states. In such cases, the NA coupling is weak, and loss of coherence occurs faster than the transition and should be accounted for.^64^ For the electron–hole (e–h) recombination process, inclusion of a decoherence effect into the simulation is necessary because coherence between band edge states is orders of magnitude shorter than the nonradiative e–h recombination time. The decoherence effect is incorporated into the e–h recombination simulation via the decoherence-induced surface-hopping (DISH) approach.^65^

All quantum-mechanical simulations, including geometry optimization, molecular dynamics simulation, and NA coupling matrix calculations, are performed using the SCC-DFTB methodology^66^ implemented in the DFTB+ software.^67^ We employed mio^66^ Slater–Koster files for carbon, hydrogen, nitrogen, and oxygen. For the zinc atom, the parameter set generated by Sarkar and his group^68^ is used. It is compatible with the mio data set for the SCC-DFTB calculations. The geometry optimization of the nanorings is followed by heating to room temperature for 6 ps utilizing velocity rescaling.^69^ Then, 3 ps MD trajectories are generated with a 1 fs time step using the Verlet algorithm.^70^ These trajectories are used to obtain adiabatic state energies and NA couplings for excited state NAMD simulations. Hot hole relaxations in the VB and ET in the CB are simulated by applying the fewest switches surface-hopping (FSSH) algorithm.^71,72^ On the contrary, the nonradiative e–h recombination process is modeled using the DISH approach^65^ that includes decoherence effects. Both surface-hopping approaches are implemented in the PYthon eXtension for Ab Initio Dynamics (PYXAID) code.^73,74^ The combined SCC-DFTB and NAMD methodology has been successfully employed in a wide range of condensed matter^29,75−82^ and molecular^4,44,45^ systems.

A schematic energy diagram of the photoexcited carrier dynamics is presented in Figure 1a. The hot hole is generated upon photoexcitation of an electron from deep inside the VB into the higher-energy CB states of the porphyrin nanorings. Then, the hot hole relaxes within the VB through energy dissipation to vibrations. On the contrary, the photogenerated electron is simultaneously cooled to the CB edge and separates from the hole by transfer to the nanoring core due to the type II alignment of the electronic states of the periphery and the core of the nanorings. The charge separation is the key initial step of the solar cell operation. Subsequently, the spatially separated exciton undergoes nonradiative recombination by relaxing to the ground electronic state via coupling to vibrations.

**Figure 1:**
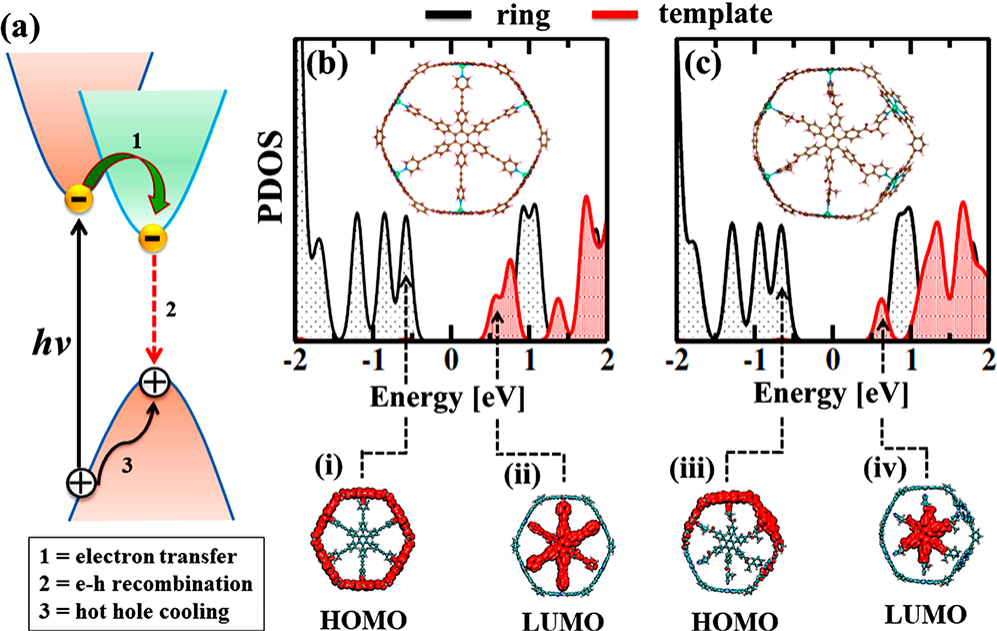
(a) Schematic of the photoexcited carrier dynamics in porphyrin nanorings. Upon photon irradiation, electrons are excited over a broad energy range, and hot holes are created. The photoexcited electron is transferred from the nanoring to the template (1), and hot holes cool by dissipating the excess energy to vibrations (3). Finally, the lowest-energy charge transfer exciton undergoes nonradiative electron–hole (e–h) recombination to relax back to the ground state (2). Projected density of states (PDOS) of the optimized geometries of (b) Z6-T6 and (c) Z6-CT6. Both systems exhibit type II band alignment with a finite energy gap. The optimized geometries of the template-directed Z6-T6 and Z6-CT6 porphyrin nanorings are shown in the insets. Carbon, hydrogen, nitrogen, oxygen, and zinc atoms are shown as brown, pink, blue, red, and green spheres, respectively. The bottom panels show HOMO and LUMO charge densities of Z6-T6 (i and ii) and Z6-CT6 (iii and iv). The charges are fully delocalized over the corresponding moieties in Z6-T6, whereas the charges are partially localized in Z6-CT6.

The optimized geometries of the systems under investigation are shown in panels b and c of Figure 1. Six porphyrin units are connected through 1,3-diethynylbenzene (*m*-phenylene) spacers at the meso positions of the porphyrin to form a cyclic porphyrin nanoring, Z6. Alongside, optically active (chiral) and optically inactive hexa-dented templates (CT6 and T6, respectively) embrace the porphyrin, holding it as “spokes in a wheel” to provide rigidity and stability. The T6 and CT6 templates fits well into the cavity of the Z6 porphyrin nanoring, forming supramolecular complexes Z6-T6 and Z6-CT6. Each central Zn atom of the nanoring is pentacoordinated, with the fifth coordination occurring through a N atom of a template’s pyridine in both systems. The Z6 core is connected with the T6 template via the acetylenic groups through the *para* position of pyridines. On the contrary, the Z6 core is connected to the *meta* position of pyridines in the CT6 template through ethyl formates. The optical activity of the CT6 template is evidenced by the circular dichroism and circularly polarized luminescence experiments.^46^

The simulated averaged gap of Z6-T6 is 1.06 eV at room temperature. Replacement of T6 with optically active CT6 extends the gap to 1.14 eV. The ultraviolet–visible (UV–vis) absorption spectra of both systems, simulated using time-dependent DFTB, exhibit absorption maxima at ∼2.75 eV (see Figure S1), consistent with the experimental absorption maximum at 450 nm (2.755 eV).^46^ To elucidate the photophysical processes initiated by photoexcitation, it is essential to focus on the electronic alignment and nature of localization of the key orbital participating in the carrier dynamics. The projected densities of states (PDOS) of the current systems are shown in panels b and c of Figure 1. Photoexcitation in the porphyrin nanoring causes ET from the Z6 core to the template (T6/CT6). At the same time, the hot hole undergoes intraband hole cooling. At the band edge, both systems possess a type II energy alignment. The HOMO is localized on the Z6 core of the porphyrin nanoring, while the LUMO is on the T6 and CT6 templates (Figure 1, i–iv). The charges in the band edge states are fully delocalized over the core and the template of Z6-T6. On the contrary, the charges are partially localized in Z6-CT6. Such localization of the wave function affects the dynamics by altering the NA coupling between the band edge states because the delocalized nature of the wave function enhances the electronic overlap.

To investigate the intraband hot hole relaxation, electrons are promoted to the CB from VB states down to 1.5 eV from the VB edge. The excited hole relaxation dynamics is considered for 11 initial states, from HOMO-1 to HOMO-11. Panels a and b of Figure 2 illustrate the dynamics of the intraband hole relaxation showing the population decay of each excited state. The population decays are fitted with a linear combination of Gaussian and exponential functions: *y* = *A* exp(−*t*/τ_exp_) + (1 – *A*) exp(−*t*/τ_gau_)_2_. The time scales reported in Table 1 are calculated as the weighted average of the exponential and Gaussian time constants: 1/τ = *A*/τ_exp_ + (1 – *A*)/*τ*_gau_. The initial hot hole relaxation exhibits Gaussian character and then becomes exponential.^24^ The Gaussian component of the quantum dynamics is the source of the quantum Zeno effect.^64,83^ The transition from the Gaussian to exponential regime occurs when the number of quantum states involved in the dynamics becomes large. The initial Gaussian nature of the hot hole relaxation dynamics is demonstrated in Figure S2. Depending on the initial energy, the hot hole decays within 0.7–2.4 ps in the Z6-T6 porphyrin nanoring, which is corroborated well by the experimental observation (τ ∼ 2.4 ps).^46^ The simulated results show that the hole cooling is slower in Z6-CT6 (0.8–2.8 ps) than in Z6-T6. Therefore, we have established that in most cases optically active CT6 reduces charge–phonon scattering and slows hot carrier cooling that occurs from the deep VB.

**Table 1:** Intraband Hot Carrier Relaxation Times (picoseconds) in the Porphyrin Nanoring Systems

hole	HOMO-1	HOMO-2	HOMO-3	HOMO-4	HOMO-5	HOMO-6	HOMO-7	HOMO-8	HOMO-9	HOMO-10	HOMO-11
Z6-T6	1.6	1.7	1.5	1.9	1.5	2.4	1.0	0.9	0.8	0.7	0.8
Z6-CT6	2.6	1.9	1.2	1.4	1.2	2.8	1.8	0.8	0.9	1.0	1.2

**Figure 2:**
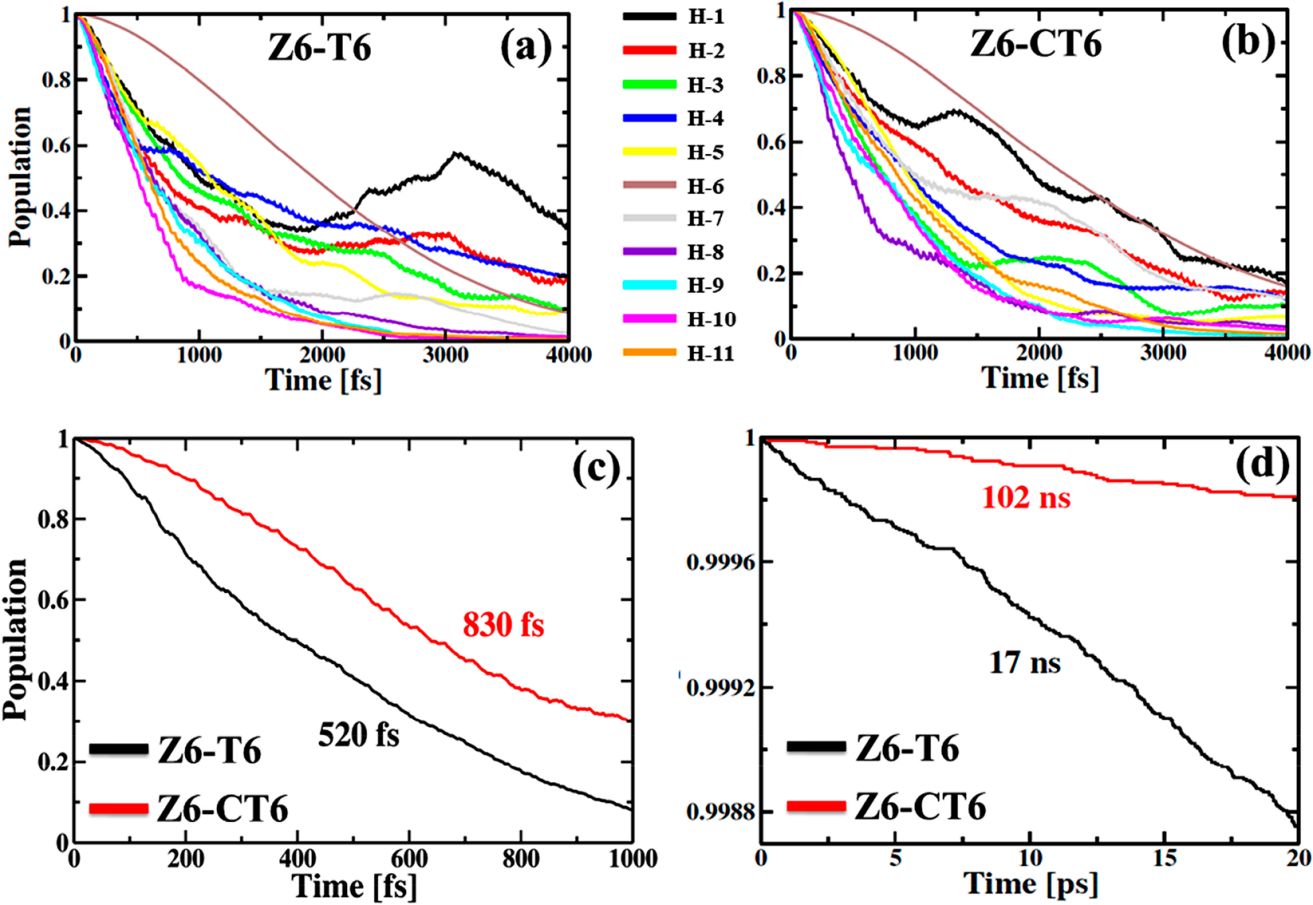
Decay of populations of hot holes during intraband relaxation in the VB in (a) Z6-T6 and (b) Z6-CT6. The time scales obtained by fitting the simulated data with combined Gaussian and exponential functions are listed in Table 1. The hot hole relaxation of Z6-CT6 is slower than that of Z6-T6. (c) Population decay of the electron donor states in Z6-T6 (black line) and Z6-CT6 (red line) during the electron transfer (ET) process. Both time scales are ultrafast. The ET is on a subpicosecond scale and is slightly slower for the Z6-CT6 system. (d) Population decay of the first excited state during the nonradiative e–h recombination of the porphyrin nanorings. The time scales are obtained by fitting the decay data with the short-time linear approximation to the exponential function. The e–h recombination is 6 times slower in Z6-CT6 than in Z6-T6.

An electron, excited in the nanoring systems from the VB to the CB, moves from Z6 to the template (T6/CT6) on a subpicosecond time scale. The decay of populations of the electron donor states is shown in panels c and d of Figure 2. The corresponding time scales are also obtained by fitting the simulated data with the combined Gaussian and exponential functions (Table 2). The ET from the photoexcited Z6 to the T6 template is ultrafast (520 fs). Replacement of T6 with CT6 slows the transfer to 830 fs. In both cases, the ET is ultrafast, leading to an efficient photoinduced charge separation.

**Table 2:** Average Energy Gaps, Root-Mean-Square Nonadiabatic (NA) Coupling, Pure-Dephasing/Decoherence Times, and Time Scales of Electron Transfer and Nonradiative e–h Recombination for the Z6-T6 and Z6-CT6 Nanorings

process	system	band offset/gap (eV)	NA coupling (meV)	decoherence (fs)	time scale (ps)
ET	Z6-T6	0.13	24.83	30	0.52
	Z6-CT6	0.10	19.22	7	0.83
ER	Z6-T6	1.06	0.22	10.1	17 000
	Z6-CT6	1.14	0.14	9.4	102 000

On a long nanosecond time scale, the separated electron and hole recombine nonradiatively, converting the system back to the ground state. In Figure 2d, the decays of the population of the first excited states are fitted with the short-time linear approximation of the exponential decay: *y* = exp(−*t*/τ) ≈ 1 – *t*/τ. A summary of the ET and e–h recombination results is provided in Table 2. The charge recombination time scale for both nanoring systems is consistent with the previous reports on analogous porphyrin systems.^84,85^ The photoexcited charge recombination takes place within 17 ns in the Z6-T6 system. Replacement of T6 with optically active CT6 results in a significantly slower relaxation. The lifetime of the charge separated excited state increases by a factor of 6 to 102 ns. Such an increase is favorable for solar energy applications.

The photogenerated carrier relaxes nonradiatively by losing excitation energy through inelastic electron–phonon scattering, guided by the NA coupling expressed as *d*_*ij*_ = ⟨*ϕ_i_*[*r*, *R*(*t*)]|∂*ϕ*_*j*_[*r*, *R*(*t*)]/∂*t*⟩, where *i* and *j* are two adiabatic states and *r* and *R* are the electronic and nuclear coordinates, respectively. Panels a and b of Figure 3 depict contour representations of the NA coupling between the states involved in hole cooling. Note that NA couplings are larger between neighboring states because the NA coupling is inversely proportional to the energy gap between the states. Overall, the NA coupling is lower in magnitude for Z6-CT6 than for Z6-T6. Thus, the smaller electron–phonon NA coupling retards the relaxation of the hole in the former case. As shown in Table 2, large NA couplings present between the donor and acceptor states drive the ET on a subpicosecond time scale in both systems. The comparatively larger NA coupling in Z6-T6 (24.83 meV), compared to that in Z6-CT6 (19.22 meV), is responsible for the faster ET. The e–h recombination is slow, in the nanosecond regime, because the energy gap for the recombination is large and the NA coupling is small. Z6-CT6 has a smaller NA coupling (0.14 meV), leading to a slower e−h recombination compared with that of Z6-T6 (0.22 meV).

**Figure 3:**
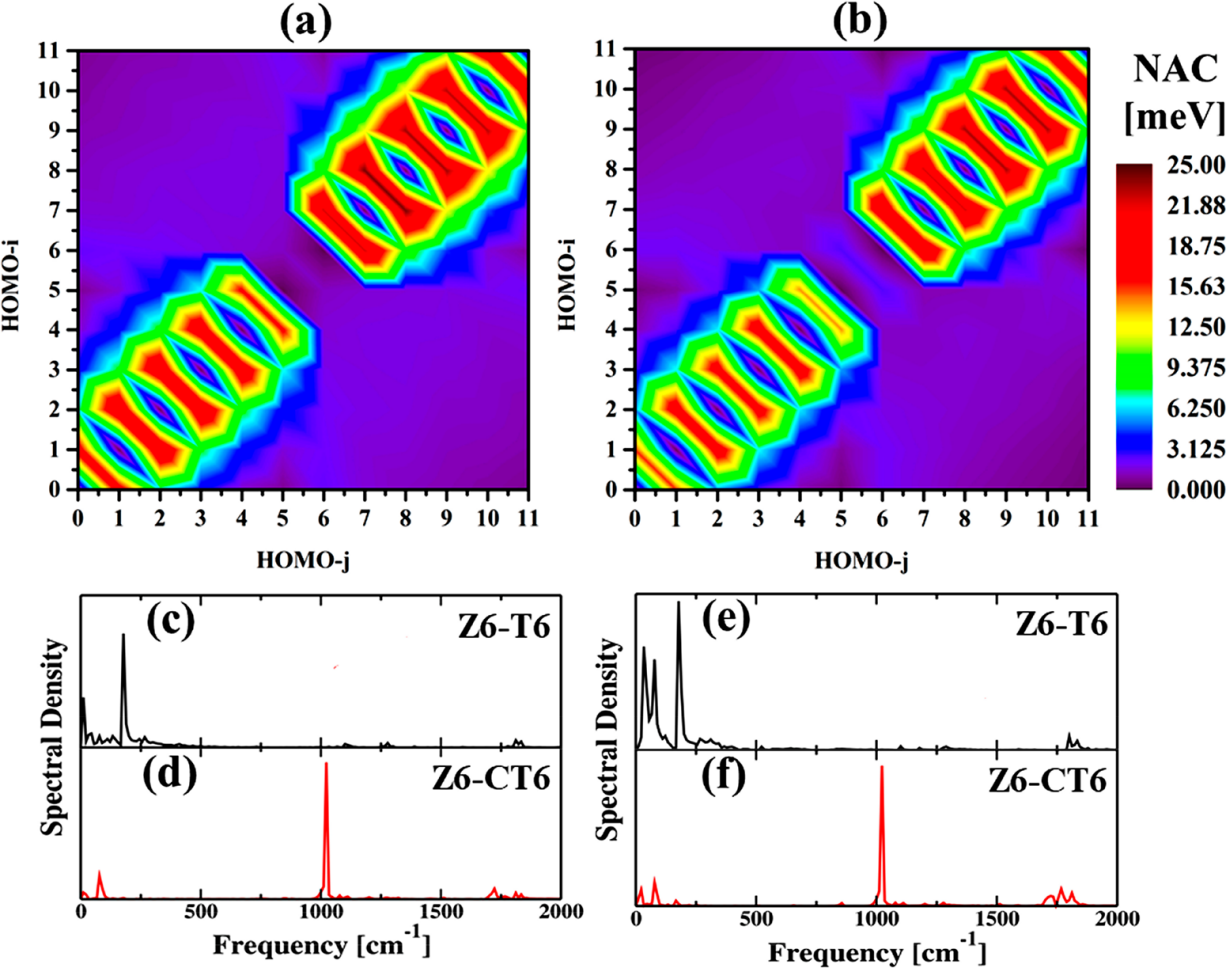
Contour representation of the root-mean-square nonadiabatic (NA) coupling between Kohn–Sham orbitals in the valence bands of (a) Z6-T6 and (b) Z6-CT6. The color bar on the right side depicts the strength of NA coupling in the contour plots. The NA coupling is stronger in Z6-T6 than in Z6-CT6. Fourier transforms (FTs) of fluctuations of energy gaps between the electron donor and acceptor states for the ET in (c) Z6-T6 and (d) Z6-CT6. Mainly low-frequency vibrational modes are involved in Z6-T6, whereas the strong phonon at ∼1020 cm^–1^ mainly drives the slower transfer in Z6-CT6. FTs of fluctuations of energy gaps between the band edge states of (e) Z6-T6 and (f) Z6-CT6 participating in the e–h recombination.

Inelastic electron–phonon scattering is responsible for electronic energy dissipation to vibrations during nonradiative carrier relaxation. The phonon modes participating in the dissipation can be identified by Fourier transforms (FTs) of the energy gaps between the initial and final states (Figure 3c–f). Figure S3 presents velocity FTs, representing all modes present of the porphyrin nanoring systems. High-intensity phonon peaks between 500 and 1500 cm^–1^ are present in the velocity FTs in both systems. The vibrational peaks below 250 cm^–1^ are assigned to the skeletal out-of-plane vibration of porphyrin, and the peaks around 500 cm^–1^ are due to breathing modes of the porphyrin.^86−88^ The phonon mode around 1020 cm^–1^ may evolve either from the in-plane asymmetric stretching of the pyrrole ring of the porphyrin^86^ or from the symmetric stretching of the C–O–C plane of the templates.^89^ Mainly strong low-frequency vibrational modes (<250 cm^–1^) are involved in the ET of Z6-T6 (Figure 3c), whereas phonon modes around 1020 cm^–1^ are predominantly involved with few low- and high-frequency modes in Z6-CT6 (Figure 3d). The strong low-frequency spectral modes in Z6-T6 arise due to the out-of-plane skeletal vibration of porphyrin, and these out-of-plane vibrations increase the number of orbital interactions between donor (fully delocalized porphyrin ring) and acceptor (fully delocalized T6) states because donor and acceptor moieties are orthogonal to the plane of the system when they are involved in out-of-plane vibrations.^90^ Thus, better orbital interactions create strong NA coupling in Z6-T6 for ET. Though the high-frequency mode (1020 cm^–1^) is involved in Z6-CT6, NA coupling is weaker for ET due to the partial delocalization of charge in both donor and acceptor states. Thus, all of the in-plane asymmetric stretching modes of the pyrrole rings in porphyrin units do not contribute to energy dissipation. Therefore, Z6-T6 possesses faster ET (520 fs) between the nanoring and templates compare to that of Z6-CT6 (830 fs). In e–h recombination, the pattern of spectral density resembles ET in both systems. Three strong low-frequency phonon modes (<250 cm^–1^) are present for Z6-T6 (Figure 3e), whereas a peak around 1020 cm^–1^ is the main contribution from Z6-CT6 (Figure 3f). The localization of charge in band edge states is similar to that in ET; the HOMO and LUMO are fully delocalized in the porphyrin nanoring and templates, respectively, in Z6-T6, whereas on Z6-CT6, the HOMO and LUMO are partially delocalized in the porphyrin nanoring and templates, respectively. For the same reason as seen for ET, out-of-plane vibrational modes increase the level of orbital overlap, which further enhances the NA coupling at the band edge in Z6-T6. Because of the partial delocalization of charge in the band edge states, the involvement of the in-plane asymmetric stretching modes of the pyrrole rings in the porphyrin units is weaker in Z6-CT6. Thus, the exciton lifetime is longer in Z6-CT6 (102 ns) than in Z6-T6 (17 ns).

Elastic electronic–vibrational interaction alters the phase relationship between the electronic wave functions of the initial and final states of a quantum transition, causing loss of coherence.^53^ We compute the decoherence function as the pure-dephasing function of the optical response theory by applying the second-order cumulant approximation.^91^ The decoherence time, *τ*_gau_, is obtained by fitting the pure-dephasing functions with a Gaussian function: *y* = *A* exp[−0.5(*t*/*τ*_gau_)^2^]. Coherence between the electron donor and acceptor states is much longer in Z6-T6 (30 fs) than in the Z6-CT6 nanoring (7 fs), as shown in Table 2 and Figure S4. Faster quantum decoherence leads to a slower intersubunit ET in the latter case. The coherence is short for the e–h recombination in both Z6-CT6 (9.4 fs) and Z6-T6 (10.1 fs), as shown in Table 2 and Figure S5. The comparatively shorter coherence retards the e–h recombination dynamics in Z6-CT6. The pure-dephasing function is related to the integral of the autocorrelation function (ACF) of the fluctuation of the energy gap between the initial and final states.^91^ The less oscillatory behavior of the ACF in the Z6-CT6 nanoring compared to that of Z6-T6 (Figure S6) leads to faster decoherence and suppressed nonradiative e–h recombination.

Charge transfer, relaxation, and recombination have been analyzed and explained by the various dynamical factors arising at ambient temperature, and the physics behind the photoinduced dynamics has been revealed by the orientation and charge localization of the photoactive states and feasibility of π-electron delocalization through templates in the porphyrin nanoring systems. In the case of intraband hot hole relaxation of the photoexcited hole, analyzing the key orbitals that participate in the dynamics is relevant. The hole states (H-1–H-5) are delocalized over the whole porphyrin nanoring in Z6-T6, but upon inclusion of the optically active CT6 in the ring, the wave functions become localized. The charge is distributed within a few porphyrin units of the nanoring. The partial localization of hole states in the Z6-CT6 system reduces the NA coupling and slows hole cooling. In the ET dynamics, the donor state (L+6) is contributed by the porphyrin π-ring, and the state is delocalized in Z6-T6. In turn, the acceptor states (L+5–L) are contributed by template T6. The electronic communication between the Z6 and T6 subsystems is favorable due to the π-resonance effect as Z6 is connected with T6 at the *para* position of pyridine with a π-acetylenic spacer (Figure S7). This facilitates orbital overlap and hybridization between Z6 and T6, as reflected by the stronger NA coupling in Z6-T6 (24.83 meV). In contrast, CT6 depletes some portion of the donor charge, creating a localized charge distribution in the L+2 state of Z6-CT6 (Figure 4), and the ET is less effective. In addition, Z6 is connected with CT6 in the *meta* position of pyridine with the ethyl formate spacer, and the electronic communication between the subsystems is hindered. The orbital overlap and hybridization between Z6 and CT6 are weakened, as reflected by the smaller NA coupling in Z6-CT6 (19.22 meV). Moreover, the number of acceptor states (L+1 and L, i.e., two) is substantially reduced compared to that for Z6-T6 (L+5–L, i.e., six). The analysis shows that ET dynamics depends on the localization of the donor and acceptor states, in particular, orbital overlap or hybridization between Z6 and the templates (T6/CT6), and the number of acceptor states.

**Figure 4:**
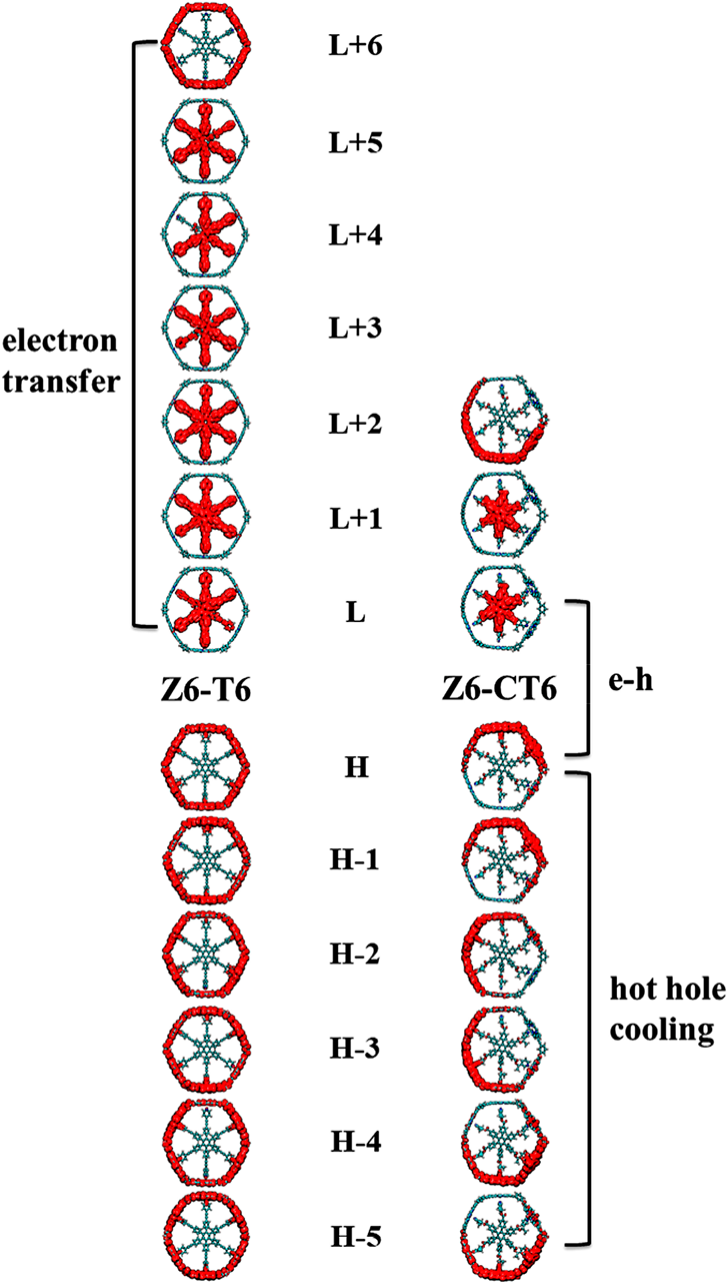
Charge densities of the key orbitals participating in carrier relaxation dynamics. The photoexcited donor states (H-1–H-5) are delocalized through the porphyrin π-ring in Z6-T6. However, upon inclusion of optically active CT6 in the ring, the wave functions become localized. The donor state (L+6) for the ET in Z6-T6 is contributed by the porphyrin π-ring, and the charge is evenly delocalized. In contrast, the CT6 template creates a localized charge distribution (L+2) in Z6-CT6. CT6 significantly depletes some π-electron density from the porphyrin π-ring, creating a localized acceptor state in Z6-CT6 for e–h recombination.

The initial state (LUMO) for e–h recombination is delocalized over the template subunit in both T6 and CT6. In comparison, the final states (HOMO) are rather dissimilar (Figure 4). The optically active CT6 significantly depletes the π-electron density from parts of the porphyrin π-ring, creating a localized acceptor state in Z6-CT6, whereas the HOMO is totally delocalized in Z6-T6. This feature extends the excited state lifetime in Z6-CT6. The reduced orbital overlap between Z6 and the CT6 template is reflected in the smaller NA coupling in Z6-CT6 (0.14 meV) compared to that in Z6-T6 (0.22 meV). The weaker NA coupling reduces the rate of e–h recombination.

In conclusion, NAMD theory combined with the SCC-DFTB method has been applied to investigate carrier relaxation, separation, and recombination in the porphyrin nanorings. The emphasis has been placed on elucidating how π-electronic conjugation in optically active templates controls charge and energy losses. The calculated intraband hot hole relaxation time scales are in good agreement with the experimental findings reporting subpicosecond to picosecond time regimes. The hot hole relaxation is slower in the optically active Z6-CT6 system because of weaker NA coupling and partial charge localization. At the same time, the photogenerated electron is transferred rapidly from the nanoring to the template in both systems, creating a long-lived charge-separated state. Strong NA coupling and a small energy offset between the donor and acceptor states, as well as involvement of out-of-plane vibrational motions, facilitate 520 and 830 fs ET for Z6-T6 and Z6-CT6, respectively. The ET is slightly slower in the Z6-CT6 system due to comparatively weaker NA coupling, partial charge localization, and shorter coherence. Most importantly, both nanorings exhibit extended lifetimes of the charge-separated states, extending into tens of nanoseconds. The exciton recombines at the band edge of Z6-T6 with a time constant of 17 ns, whereas the optically active CT6 template slows the recombination by a factor of 6, to 102 ns. The slower charge recombination induced by the optically active template is rationalized by several factors. The overlap between the electron and hole wave functions is reduced, which, in turn, weakens the NA coupling from 0.22 meV in Z6-T6 to 0.14 meV in Z6-CT6. The canonically averaged energy gas is increased by 0.1 eV from 1.06 eV in Z6-T6 to 1.14 eV in Z6-CT6. More phonon modes participate in charge recombination in the optically inactive system than in the optically active system. Finally, the coherence at the band edge is shorter in the presence of the optically active template. All of these factors collectively suppress the charge recombination in the Z6-CT6 nanoring. The systematic time-domain atomistic investigation of the nonradiative intraband carrier relaxation, charge transfer, and nonradiative e–h recombination phenomena in the porphyrin nanoring systems mimics most closely the time-resolved experiments and rationalizes how modest structural changes can cause significant changes in photophysical properties. The established detailed mechanism of control of the charge carrier dynamics by optically active templates provides valuable guidelines for the development of efficient solar energy and optoelectronic devices.

## Supporting Information Available

UV–vis spectra from the TD-DFTB approach, initial stage of hot hole relaxation dynamics, Fourier transforms of velocity autocorrelation functions, pure-dephasing functions for electron transfer and nonradiative e–h recombination, autocorrelation functions of energy gap fluctuations, and schematics of π-electron delocalization between the nanorings and the templates in the porphyrin macromolecular systems (PDF)
