# Supplementary material for: Controlling Charge Carrier Dynamics in Porphyrin Nanorings by Optically Active Templates
Source: J Phys Chem Lett. 2023 Dec 11;14(50):11384–92. doi: 10.1021/acs.jpclett.3c03304 (PMC10749466; doi:10.1021/acs.jpclett.3c03304)
Supplement: Supplementary file 1 — jz3c03304_si_001.pdf [file jz3c03304_si_001.pdf]

Supplementary Information for

Controlling Charge Carrier Dynamics in Porphyrin Nanorings by Optically Active Templates

Shrabanti Mondal,[1] Uttam Chowdhury,[1] Subhajit Dey,[1] Md Habib,[1,2] Carlos Mora Perez,[3] Thomas Frauenheim,[4,5,6] Ritabrata Sarkar,[1,4*] Sougata Pal,[1*] and Oleg V. Prezhdo[3,7*]

[1]Department of Chemistry, University of Gour Banga, Malda 732103, India

[2]Department of Chemistry, Sripat Singh College, Jiaganj 742122, India

[3]Department of Chemistry, University of Southern California, Los Angeles, CA 90089, USA

[4]Bremen Center for Computational Materials Science, Universität Bremen, Bremen 28359, Germany

[5]Beijing Computational Science Research Center, Beijing 100193, China

[6]Shenzhen JL Computational Science and Applied Research Institute, Shenzhen 518109, China

[7]Department of Physics and Astronomy, University of Southern California, Los Angeles, CA 90089, USA

*Corresponding authors, e-mail addresses: ritabratasarkar90@gmail.com, sougatapal_1979@yahoo.co.in, prezhdo@usc.edu.

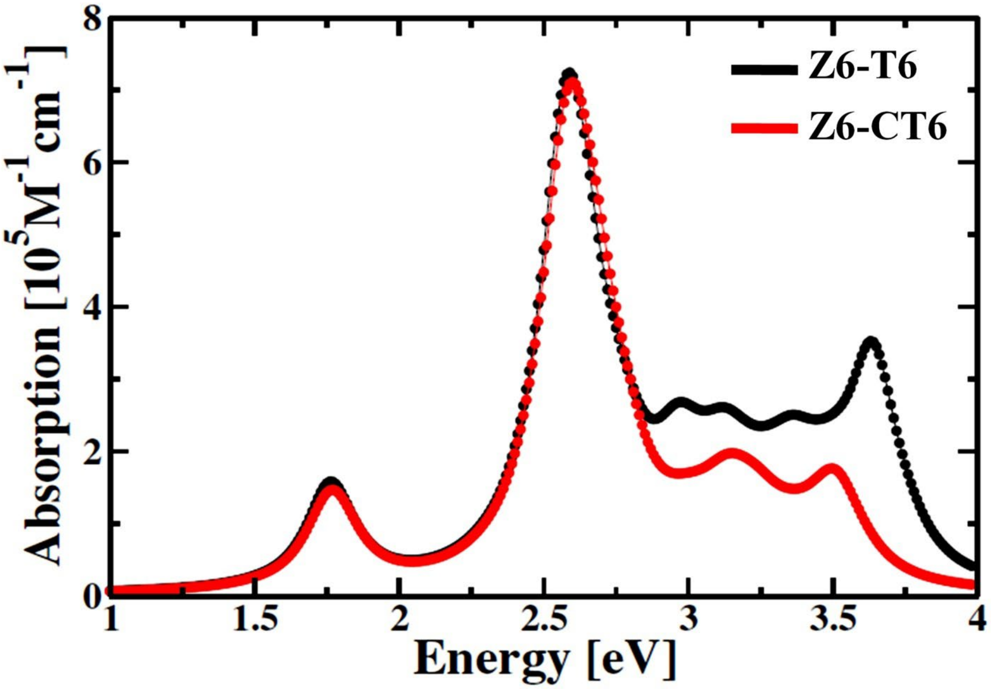

Figure S1. *UV-Vis absorption spectra of the two systems simulated using TD-DFTB. Both spectra exhibit absorption maxima at ~2.75 eV, consistent with the experimental absorption maxima at 450 nm (2.755 eV).*

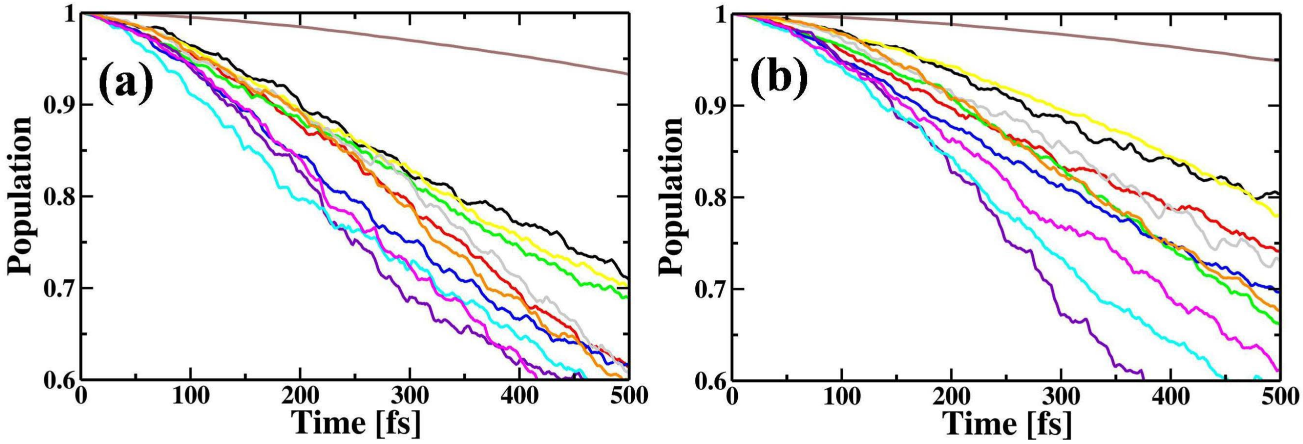

Figure S2. *Population decay of hot holes in the valence band for the first 500 fs in (a) Z6-T6 and (b) Z6-CT6. Initially, the populations exhibit Gaussian decay.*

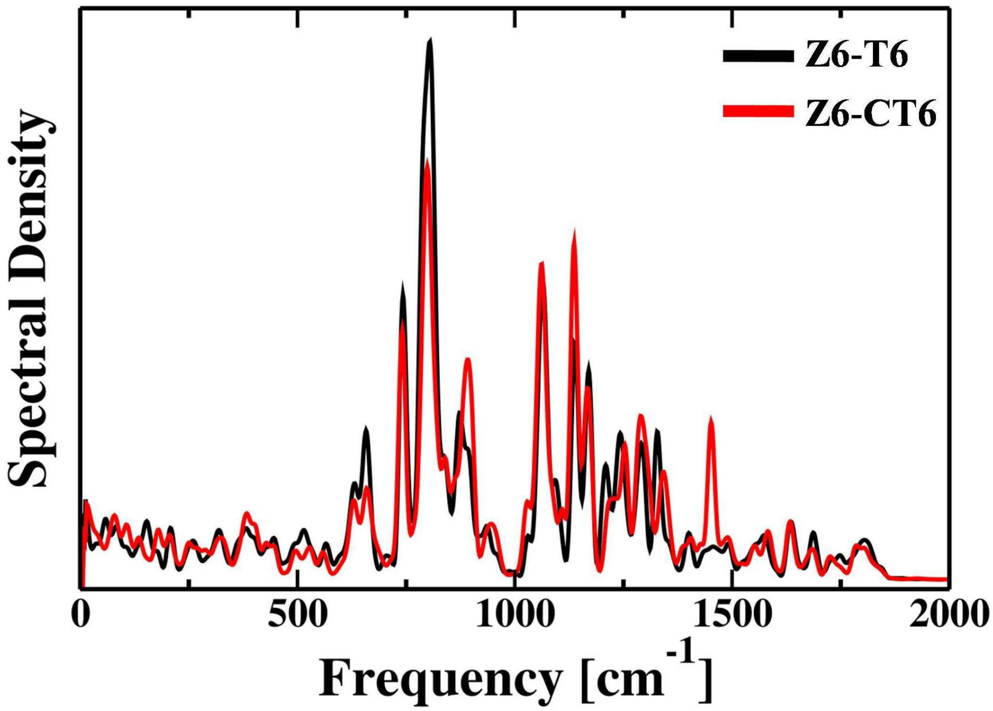

Figure S3. *Fourier Transforms (FTs) of the velocity autocorrelation function of the nanoring systems, helping to identify phonon modes participating in the excited state electron-vibrational dynamics. The velocity FT identifies all modes present in the systems. Not all of them couple to the electronic states, Figure 3.*

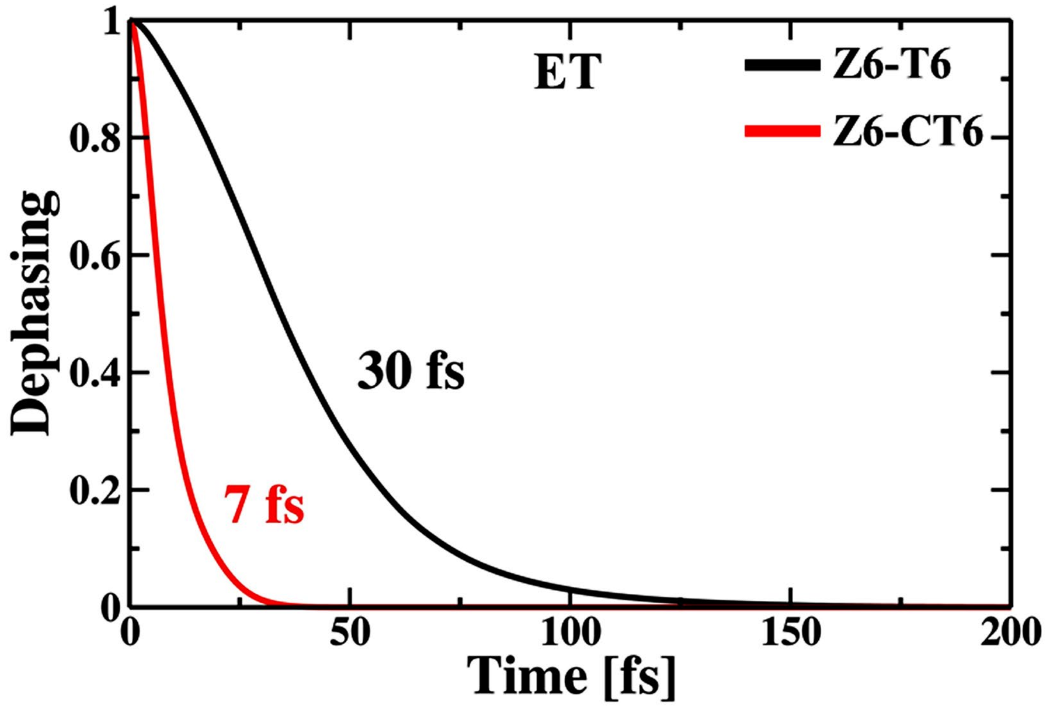

Figure S4. *Pure-dephasing functions for electron transfer in Z6-T6 (black) and Z6-CT6 (red). The pure-dephasing times,* τ_{gau}*, are obtained by fitting these functions to Gaussians,* $y = A\exp\left(-0.5\left[t/\tau_{gau}\right]^2\right)$.

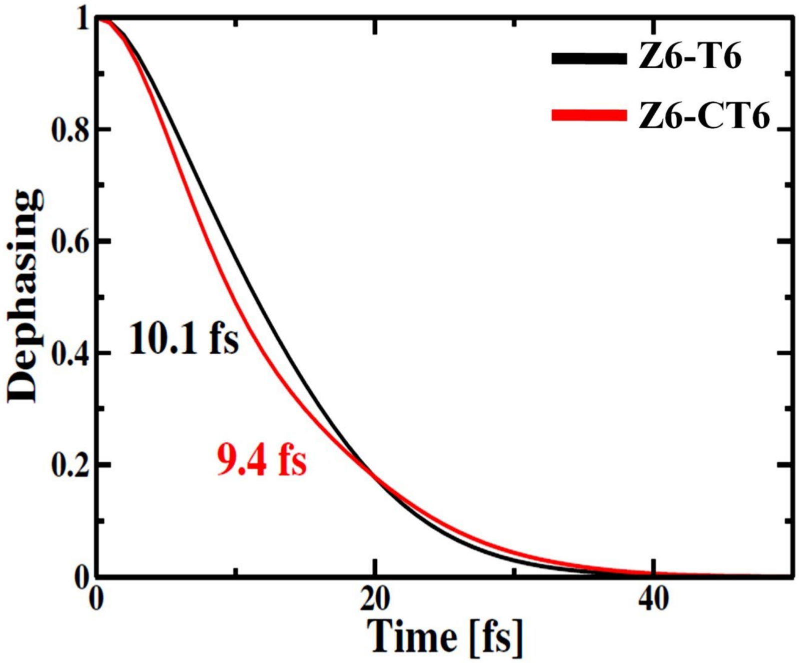

Figure S5. *Pure-dephasing functions for –e-h recombination in Z6-T6 (black) and Z6-CT6 (red). The pure dephasing times,* τ_{gau}, *are obtained by fitting these functions to Gaussians,* $y = A\exp\left(-0.5\left[t/\tau_{gau}\right]^2\right)$.

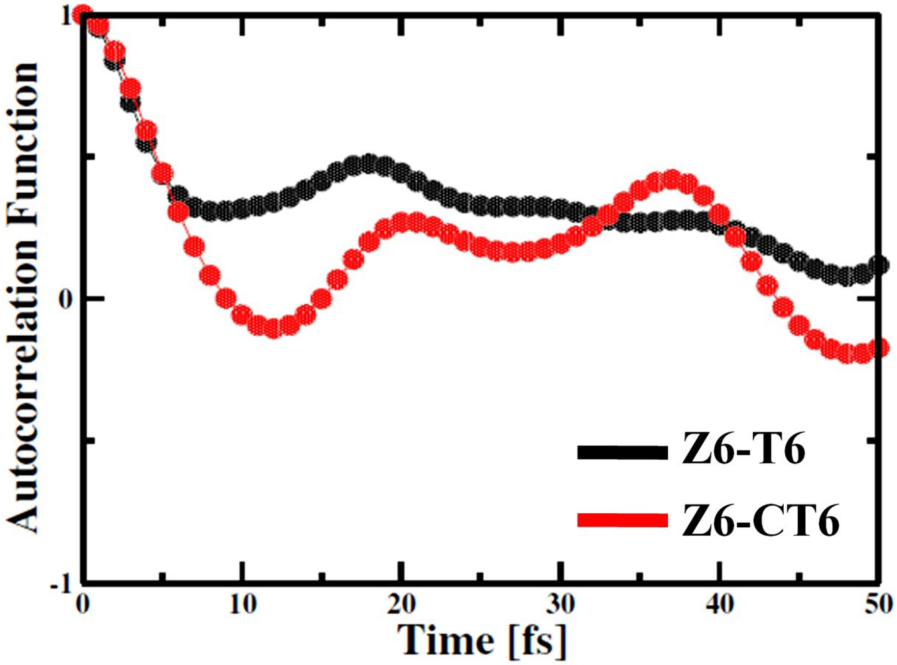

Figure S6. *Normalized auto correlation functions of the energy gap fluctuation between the band edge states in the two systems. The Z6-CT6 nanoring (red line) exhibits a larger amplitude initial, fast component and more pronounced subsequent oscillations, compared to Z6-T6 (black line).*

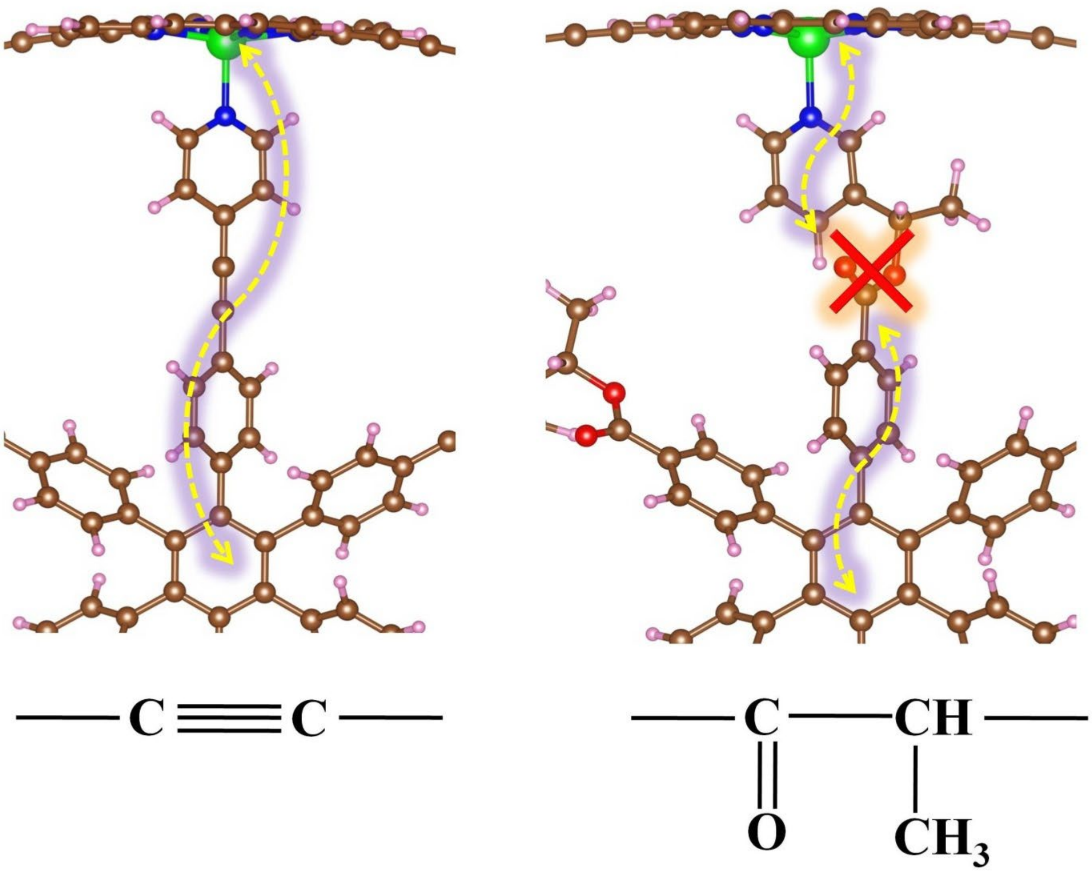

Figure S7. *The electronic communication between Z6 and T6 (only a part is shown for clarity) is favorable due to the resonance effect, as Z6 is connected to T6 at the para-position of pyridine with the π-acetylenic spacer (left panel), whereas Z6 is connected to CT6 at the meta-position of pyridine with the ethyl formate spacer, and the electronic communication is likely to be hindered in Z6-CT6 (right panel).*
